# Alumino-Silicate Structural Formation during Alkali-Activation of Metakaolin: In-Situ and Ex-Situ ATR-FTIR Studies

**DOI:** 10.3390/ma16030985

**Published:** 2023-01-20

**Authors:** Sujitra Onutai, Takeshi Osugi, Tomoyuki Sone

**Affiliations:** Nuclear Fuel Cycle Engineering Laboratories, Japan Atomic Energy Agency, 4-33 Muramatsu, Tokai-mura, Naka-gun, Ibaraki 319-1194, Japan

**Keywords:** alkali-activation, alkali-activated materials, attenuated total reflectance, structural formation, mechanisms

## Abstract

Attenuated total reflectance-Fourier transform infrared (ATR-FTIR) spectroscopy was used to demonstrate the reaction mechanisms of alkali-activated materials (AAMs) and the early stage of structure formation in the materials. The effects of different types of alkali activator solutions on the structure formation and reaction mechanisms of AAMs were studied. The results revealed that the main peaks of the ATR-FTIR spectra of the AAMs in the 1300–650 cm^−1^ range shifted to a low wavenumber with changing patterns, depending on the activator solution used, indicating that the dissolution and reorientation of metakaolin had occurred. Silica and alumina monomers were dissolved by the NaOH solution to produce crystalline zeolites. Although the reaction between metakaolin and Na_2_SiO_3_ solution is slow, the condensation between the Al-OH from metakaolin and the Si-OH from Na_2_SiO_3_ solution bonded the chain to be longer. Therefore, the Na_2_SiO_3_ solution acted as a template-bonded monomer, formed long chains of Si–O–Si and Si–O–Al, and produced an amorphous AAM structure. In the mixed solution, when the NaOH in it dissolved the Si and Al monomers, the Na_2_SiO_3_ in the solution also bonded with monomers and produced a complex structure. The different reaction that metakaolin had with different alkali activator solutions reflected the different phases, microstructures, and mechanical properties of the AAMs produced.

## 1. Introduction

Alkali-activated materials (AAMs) are becoming increasingly popular due to their excellent physical and chemical properties and they have become a valuable technology for recycling waste in the future. AAMs are inorganic polymers created through a chemical reaction between a solid aluminosilicate precursor and an alkaline activator at 25 °C or at an elevated temperature (40 °C–80 °C) [1,2]. The raw materials used to synthesize AAMs contain natural minerals [3], such as metakaolin, and industrial waste, such as fly ash, slag, rice husk ash, and aluminum hydroxide [4,5,6,7]. Metakaolin is a primary raw material (natural mineral) containing silica and alumina and suitable for AAMs. Moreover, it is a purer, more easily defined starting material, consistent chemical composition, and predictable properties. Therefore, metakaolin is widely used for industrial and research purposes. Sodium hydroxide, sodium silicate, potassium hydroxide, and potassium silicate solutions are commonly used as alkali activators [8]. The properties of an AAM are related to its structure, formed of chains of tetrahedral SiO_4_ and AlO_4_ bridged with oxygen. The Si–O–Si and Si–O–Al chains in the AAMs form three-dimensional networks as a result of the polymerization reaction [9]. While amorphous aluminosilicate is the main product of the polymerization reaction, crystalline substances, such as calcite and zeolite can also be found in AAMs [10]. As already mentioned, AAMs are made from different types of raw materials and alkali activator solutions with different parameters, such as the alkali solution concentration and the Al/Si ratio, and all the factors are related to the reaction rate, reaction mechanism, and properties of the product [11,12,13].

The reaction that occurs between the alkali activator solution and aluminosilicate raw material has several stages: dissolution, reorganization, condensation, crystallization, and hardening [11]. Generally, the two processes of dissolution of silica and alumina to form a precursor and the rearrangement of the network overlap and occur in parallel [8,14]. To study the structure and bonding of materials, techniques, such as nuclear magnetic resonance (NMR) [15] and X-ray photoelectron spectroscopy (XPS), can be used [16]. Both in-situ and ex-situ, NMR and XPS techniques have limitations: sample preparation and measurement methods are complex. A simple technique that is now being widely used to observe basic stretching and bending vibration bands in AAMs is attenuated total reflectance-Fourier transform infrared (ATR-FTIR) spectroscopy [17]. In-situ and ex-situ ATR-FTIR techniques can provide information on the distinct effects of the alkali activator solutions on the structure formation in AAM. ATR-FTIR has been used to study the early stages of structure formation in fly ash AAMs. The reaction processes of two types of AAMs formulated using fly ash, sodium aluminate or amorphous alumina, and alkali solutions were investigated [18]. Different rates of alumina release and formation were observed by the changes caused to the intensity bands of Al–O, Si–O–Si, and Si–O–Al at 710, 1100, and 950–960 cm^−1^, respectively. However, studies on the early stages of structure formation in metakaolin-based AAMs are scarce; thus, a thorough understanding of the chemistry responsible for the structure formation and hardening of alkali-activated metakaolin and other raw materials is required.

A simplified in-situ ATR-FTIR method was used to observe the early stage of the reaction mechanism of metakaolin AAM paste. A set of samples were used to display in detail the potential of the technique and obtain an insight into the processes that occur when metakaolin reacts with alkali solutions. The effect of the SiO_2_/Al_2_O_3_ ratio of the alkali solution on the reaction mechanism of AAMs was studied by using in-situ and ex-situ ATR-FTIR methods. The alkali-activated products obtained were examined using X-ray diffraction (XRD) and Rietveld analyses. The microstructures of the products were observed using scanning electron microscopy (SEM) and energy dispersive X-ray (EDX) spectroscopy. The mechanical properties of the products were analyzed by testing their compressive strength, and calculating their bulk densities. The in-situ and ex-situ ATR-FTIR techniques could be extended to analyze complex AAM forming systems.

## 2. Materials and Methods

### 2.1. Raw Materials

Metakaolin (MetaMax^®^) with a Si/Al molar ratio of 1.00, obtained from BASF Japan Ltd., Tokyo, Japan, was used as the starting material in the preparation of the AAM samples. Its composition was determined using X-ray fluorescence analysis. It contained 52.52 wt% SiO_2_, 44.34 wt% Al_2_O_3_, 1.36 wt% TiO_2_, 0.25 wt% Fe_2_O_3_, 0.05 wt% CaO, 0.20 wt% Na_2_O, and 1.28 wt% others. For alkali activation, solutions of sodium hydroxide (NaOH), sodium silicate (Na_2_SiO_3_), and a mixed solution of NaOH and Na_2_SiO_3_ were used. The Si/Al ratio of the alkali solutions was between 1.77 and 2.26. The mole and liquid/solid ratios of the mixed solution-based, NaOH solution-based, and Na_2_SiO_3_ solution-based AAMs are presented in Table 1.

### 2.2. Sample Preparation

Reagent-grade sodium hydroxide pellets (FUJIFILM Wako Pure Chemical Corporation, Osaka, Japan) and a sodium silicate solution (Nippon Chemicals Industrial, Tokyo, Japan) with 36.81 wt% SiO_2_ and 17.60 wt% Na_2_O were two of the alkali activators used. The two solutions were stored for 24 h at a controlled room temperature of 20 ± 2 °C and then mixed to obtain a mixed solution for use as the third activator solution. Metakaolin and each of the alkali activators were mixed in a mechanical mixing machine for 10 min at 20 °C. The prepared AAM pastes were then poured into a silicone cubic mold and stored in an incubator at 20 ± 2 °C until they were ready for testing.

### 2.3. Characterizations

An ATR-FTIR instrument (FTIR−4200; Jasco Inc., Tokyo, Japan, 2016) was used to analyze the bonding transformation that occurred during AAM paste polymerization. The AAM paste to be tested was placed on the diamond ATR cell. The cell was covered with Teflon tape to prevent water in the paste from leaving and control the temperature of the paste. Spectra at room temperature were first obtained at 5 min intervals within 5 to 160 min after the reaction, and then at 30 min intervals, up to 24 h of reaction. All spectra were obtained in the wavenumber range of 4000 to 400 cm^−1^ with 128 scans and a spectral resolution of 4 cm^−1^. The obtained spectra were deconvoluted using OriginPro 2021 (OriginLab Corporation, Northampton, MA, USA), and the multiple peak fit tool with the Gaussian fitting function was used (no parameters were fixed and R-square > 0.999). 

The XRD and FTIR analysis of the AAM samples were performed after they were cured at 20 °C for 28 days. The samples were first crushed and sieved to obtain a powder with particle sizes less than 106 μm. The powder was then immersed in acetone to stop any reactions and placed in a vacuum desiccator for drying [19]. The powder obtained from each AAM sample was then placed in the diamond ATR-FTIR cell, and 128 scans were performed within the test range 4000–400 cm^−1^ at a spectral resolution of 4 cm^−1^. To characterize the phase of the AAM samples, XRD was performed at a scanning step of 0.02°, in the 2θ = 5–80 range using CuKα radiation at a 40-kV tube voltage and a 20-mA tube current (Ultima IV; Rigaku, Japan, 2007). The weight fraction of the phases was determined by adding 10 wt% corundum (Alfa Aesar, Karlsruhe, Germany) to the AAM powder and performing a Rietveld analysis (SiroQuant XRD software). The microstructures and morphologies of the AAM samples were observed using SEM (JSM-6010LA; JEOL, Japan, 2011). The elemental compositions of the samples were characterized using EDX analysis. The samples were first crushed and then placed on the SEM sample stubs using carbon tape. Following the desired curing time of the samples, the compressive strength of each sample was measured and its bulk density calculated to determine its mechanical properties. For the compressive strength test, an automatic compression testing apparatus (Hi-ACTIS-200L; MARUI & Co., Ltd., Osaka, Japan, 2011) was used with a compression loading speed of 0.6 MPa/s. The bulk densities of the samples were determined by calculating their mass to volume ratios.

## 3. Results

### 3.1. ATR-FTIR Analysis

#### 3.1.1. AAM Paste

**Figure 1:**
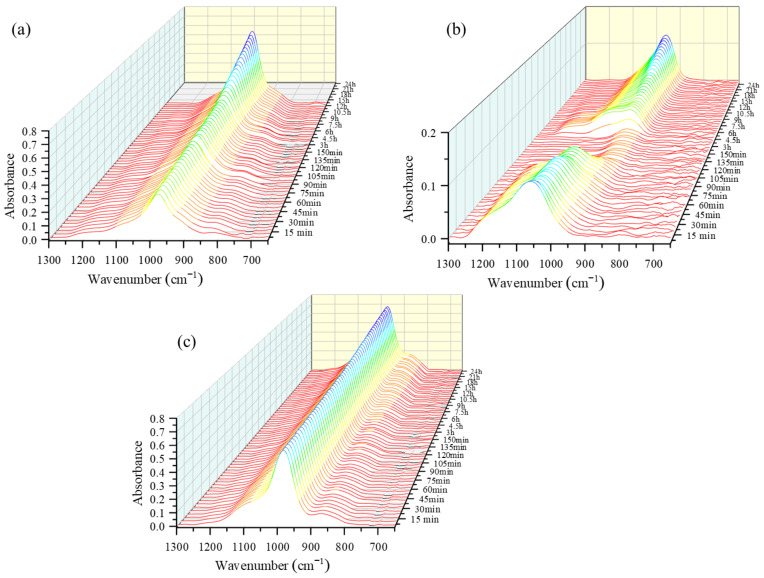
Three-dimensional waterfall attenuated total reflectance-Fourier transform infrared (ATR-FTIR) spectra of (**a**) mixed solution-based alkali activated material (AAM), (**b**) NaOH solution-based AAM and (**c**) Na_2_SiO_3_ solution-based AAM, at different time points.

**Figure 2:**
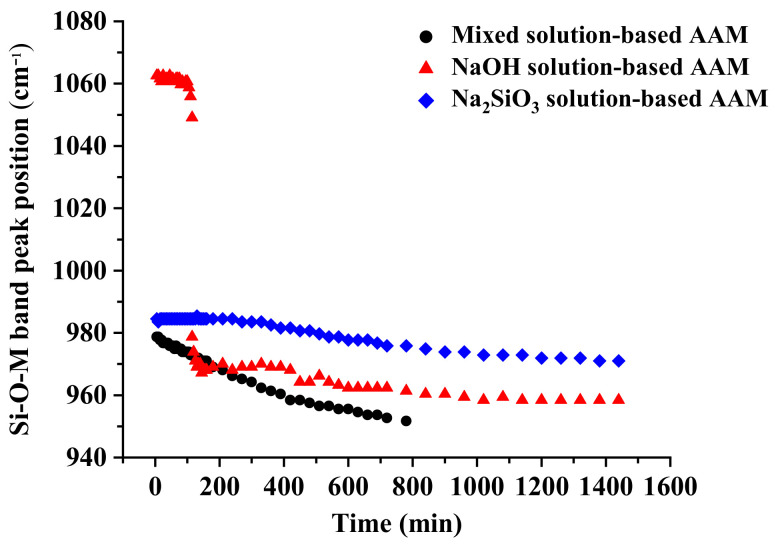
Shifting of the position of the peak of the Si–O–M position as a function of time (M = Si, Al, or Na).

**Figure 3:**
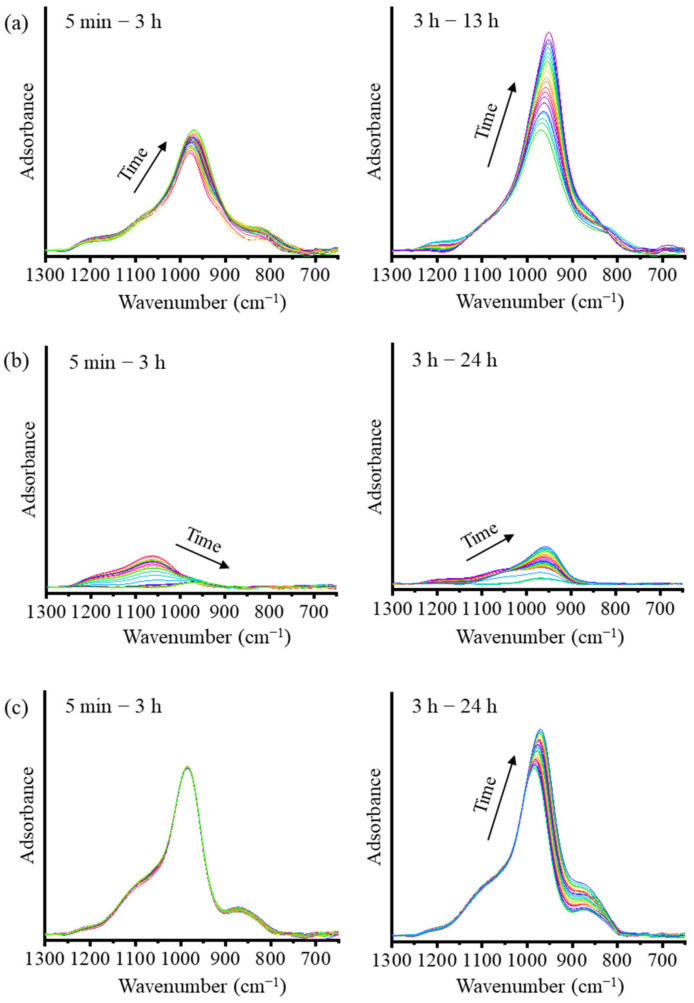
ATR-FTIR spectra of (**a**) the mixed solution-based AAM, (**b**) the NaOH solution-based AAM, and (**c**) the Na_2_SiO_3_ solution-based AAM, at different time points.

**Figure 4:**
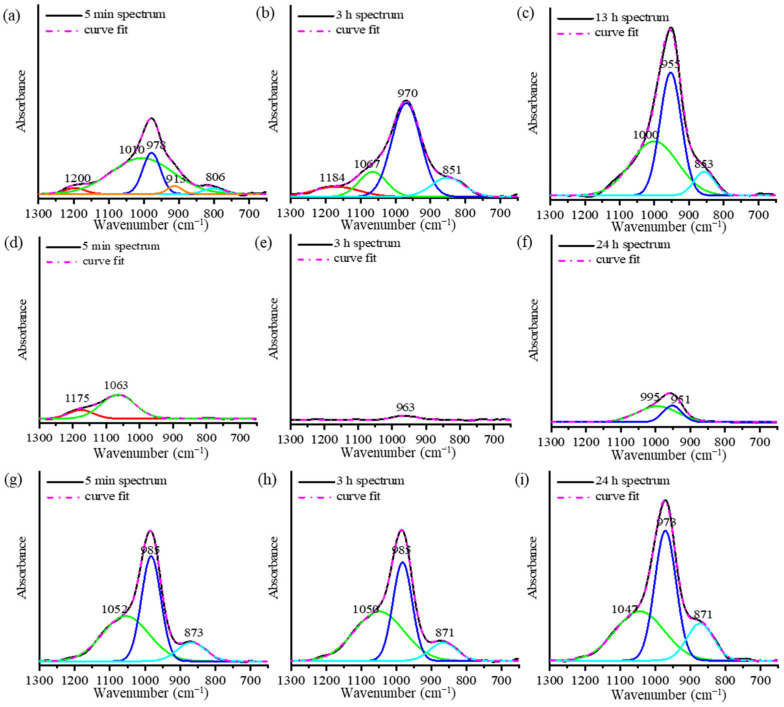
Deconvolution of the ATR-FTIR peaks of (**a**–**c**) the mixed solution-based AAM, (**d**–**f**) the NaOH solution-based AAM, and (**g**–**i**) the Na_2_SiO_3_ solution-based AAM, at different time points.

The ATR-FTIR method is a simple analytical method that can be used to study material reactions. The influence of different kinds of alkali activators on the reaction mechanisms and chemical structure of the AAMs were analyzed using ATR-FTIR spectroscopy. The ATR-FTIR spectra of the mixed solution-based AAM, NaOH solution-based AAM, and Na_2_SiO_3_ solution-based AAM, for different time points, are shown in Figure 1, Figure 2, Figure 3 and Figure 4, which have used the same scale for the intensity. Spectra were first recorded at 5 min intervals for 180 min; after that, they were recorded at 30 min intervals for the remainder of the 24-h period. The observation had to be stopped when the AAM pastes hardened because the spectra could no longer be detected. The main band observed in the 1300–650 cm^−1^ region could be attributed to the asymmetric stretching vibration of Si–O–M (M=Si, Al, or alkali ions) linkages [20]. Thus, this band in the 1300–650 cm^−1^ region was studied to explore how the linkages in the AAM pastes change during the reactions.

The three-dimensional waterfall ATR-FTIR spectra of the mixed solution-based, NaOH solution-based, and Na_2_SiO_3_ solution-based AAM pastes are presented in Figure 1. The spectra show the changes that occurred in the overall and main peak intensities due to the polymerization reaction of the AAM paste that occurred over a period of 24 h. As can be seen in Figure 1a, the peak slightly shifts to a lower wavenumber and increases in height with the increase in the reaction time. The height of the peak shoulder at approximately 800 cm^−1^ has slightly decreased. Figure 1b shows that the height of the peak of the NaOH solution-based paste at 1100 cm^−1^ decreases until 3 h, after which it starts to increase again. The heights of the peak and the peak shoulder of the Na_2_SiO_3_ solution-based AAM paste at 1000 cm^−1^ and 850 cm^−1^, respectively, increase with the increasing reaction time (Figure 1c).

In Figure 2, the position of the main peak of the Si–O–M band is displayed as a function of time. The figure shows that the peaks of the Si–O–M bands in the AAM pastes have shifted to a lower wavenumber, which could be attributed to the replacement of Si–O–Si bonds by Si–O–Al and/or Si–O–Na bonds [21]. The kinetics of this reaction could be estimated using the slope of the curve; a low slope indicates slow kinetics [22]. The behaviors of the mixed solution-based AAM and the Na_2_SiO_3_ solution-based AAM were similar; however, the mixed solution-based AAM reacted more quickly than the Na_2_SiO_3_ solution-based AAM. The Si–O–M bond signals of the mixed solution-based AAM shifted to a lower wavenumber (from approximately 980 to 950 cm^−1^) after 780 min (13 h). The peak of the Na_2_SiO_3_ solution-based AAM also shifted to a lower position (from 985 to 970 cm^−1^) after 1140 min (24 h). In the spectra of the NaOH solution-based AAM, the peak in the Si–O–M band has shifted two times. First, the peak shifted from 1060 to 1050 cm^−1^ 115 min after the reaction. Thereafter, it suddenly shifted to 980 cm^−1^ and continued to move toward lower wavenumbers. This result indicates that the NaOH solution has had a strong reaction with metakaolin at approximately 2 h after the reaction.

To understand the reaction mechanisms of the AAMs using ATR-FTIR spectra, the spectra are presented in two stages, as shown in Figure 3. The deconvolution of the main band using the Gaussian function is presented Figure 4. The main FTIR stretching vibrational bands of metakaolin-based AAMs are presented in Table 2 [20,23]. In the first stage of the analysis of the mixed solution-based AAM paste from 5 min to 3 h (Figure 3a and Figure 4a,b), after the reaction started, the height of the peak at 978 cm^−1^ is found to increase with a slight shift toward the lower wavenumber 970 cm^−1^. The deconvoluted FTIR peaks of the Si–O–Si and Al–O–Si bonds at 1200 cm^−1^ continue to appear after 5 min and 3 h of the reaction. In the second stage, between 3 and 24 h (Figure 4c), the peak shoulder at 1200 cm^−1^ decreases in height and disappears after 3 h of the reaction. The peaks at 1067, 970, and 850 cm^−1^ shift to lower wavenumbers and their heights continue to increase. This result indicates that Si–O stretching with alkali ions and OH increased as the reaction time increased. In the case of the NaOH solution-based AAM paste (Figure 3b), the decrease in the peak and the shifting of the peak to lower wavenumbers between 1200 and 1000 cm^−1^, occurred after 5 min to 3 h of the reaction. Then, the peak at approximately 960 cm^−1^ started to increase until the reaction occurred over a period of 24 h. The deconvoluted peaks of the NaOH solution-based AAM paste at 5 min, 3 h, and 24 h of the reaction are presented in Figure 4d–f. Then, after 5 min of the reaction, peaks at 1175 and 1063 cm^−1^ can be observed with the asymmetric stretching of Si–O–Si and Al–O–Si. Then, both peaks at 1175 and 1063 cm^−1^ disappear and a single peak at 963 cm^−1^, which is caused by Si–O–Na bonding, can be seen after 3 h of the reaction. Then, at 24 h, the deconvoluted peaks at 951 and 995 cm^−1^, which are due to Si–O–Na, Si–O–Si, and Al–O–Si, increase. Thus, the NaOH solution would have separated silica and alumina from metakaolin, after which Na ions would have reacted and organized with silica and alumina precursors. The bands of the Na_2_SiO_3_ solution-based AAM paste (Figure 3c) did not change during the first 5 min–3 h after the reaction. Three hours later, the bands at 985 and 871 cm^−1^ increase with the increase in the reaction time. The peaks can be confirmed by the deconvoluted peak of the Na_2_SiO_3_ solution-based AAM paste, as shown in Figure 4g–i. Between 5 min and 3 h, the peaks at 1052, 985, and 871 cm^−1^ do not shift, and the peaks at 985 and 871 cm^−1^ increase from 3 h to 24 h. The findings indicate that the Na_2_SiO_3_ solution does not efficiently dissolve the raw materials used to prepare AAMs. However, the Na_2_SiO_3_ solution can bond and react with the precursors to form long chains.

#### 3.1.2. AAM Powder

**Figure 5:**
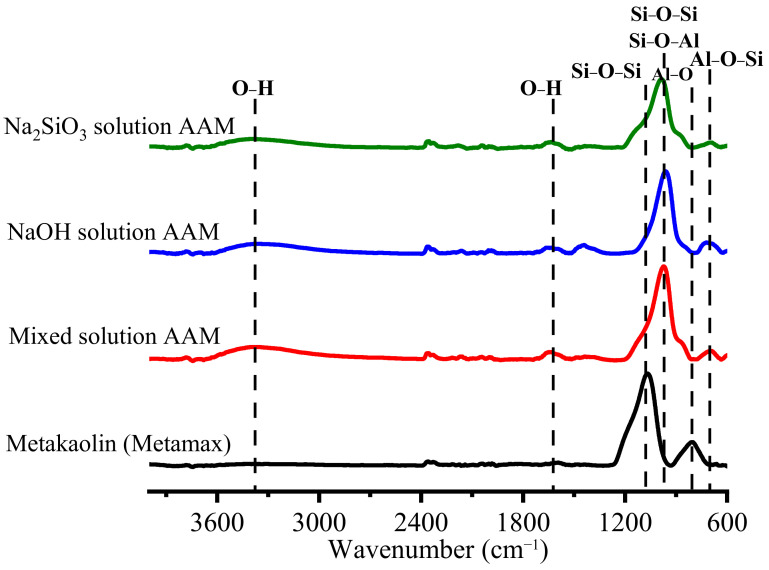
FTIR peaks of metakaolin and AAMs with different alkali activator powders.

**Figure 6:**
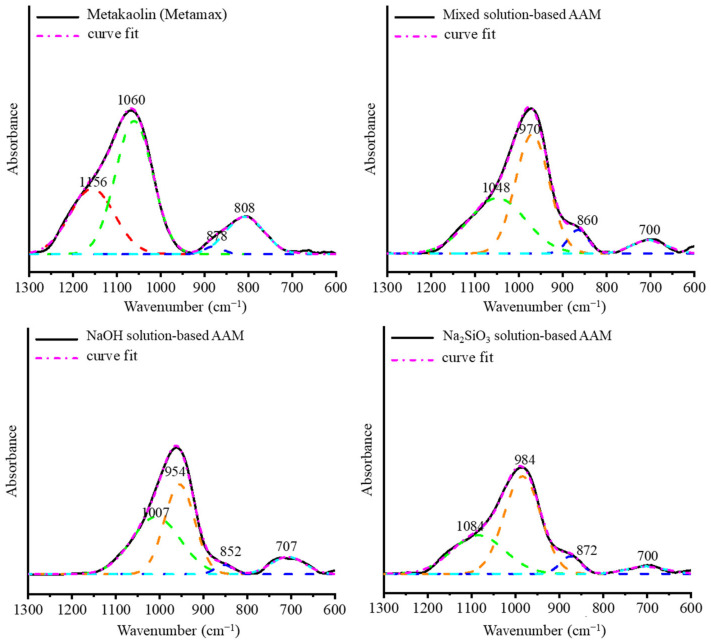
Deconvolution of the main band (1300–600 cm^−1^) of metakaolin and AAM powders with different alkali activators.

Once the AAM samples were cured at 20 °C for 28 days, they were crushed and sieved, and the reaction was stopped. As Figure 5 shows, the obtained AAM powders were tested using ATR-FTIR spectroscopy to observe their bonding after 28 days. The O–H bending and stretching vibrations were observed at 3450 and 1650 cm^−1^ [24,25]. The Si–O–Si and Si–O–Al bonds of metakaolin were detected at approximately 1100 cm^−1^. Al–O was present in metakaolin at approximately 800 cm^−1^ [20]. Once metakaolin was mixed with an alkali solution, the main peaks of the Si–O–Si and Si–O–Al bonds shifted to shorter wavenumbers between 1000 and 950 cm^−1^ and the Al–O peak disappeared. This result indicates that Si–O–Si changed to Si–O–Al because Si–O–Si bridges generate a band at approximately 1100 cm^−1^, while Si–O–Al bridges generate a band at approximately 1000 cm^−1^ [26]. In addition, in all of the AAM samples, a new peak attributed to the Si–O–Al bonding was detected at approximately 700 cm^−1^. A new peak, corresponding to a sodium carbonate band, was detected in the NaOH-based AAM powder at approximately 1460 cm^−1^ [24]. Figure 6 shows the deconvoluted peaks of the main band (1300–600 cm^−1^) of metakaolin and the samples. The bands at 1156, 1060, 878, and 808 cm^−1^ are associated with the stretching and bending vibration modes of metakaolin. Once the metakaolin was mixed with an alkali-activated solution, the peaks at 1156 and 808 cm^−1^ disappeared, and the peak at 1060 cm^−1^ was reduced. The weak band at 1200–1156 cm^−1^ in the metakaolin spectrum indicated the presence of a small amount of uncalcined kaolin in the raw material [27]. Nevertheless, the peak in the 852–878 cm^−1^ wavenumber range increased, and new peaks appeared at 970–950 cm^−1^ and 700 cm^−1^. These results confirm that metakaolin can be dissolved in alkali activator solutions and that the reorientation of its bonding would occur after 24 h to 28 days.

### 3.2. XRD Analysis

**Figure 7:**
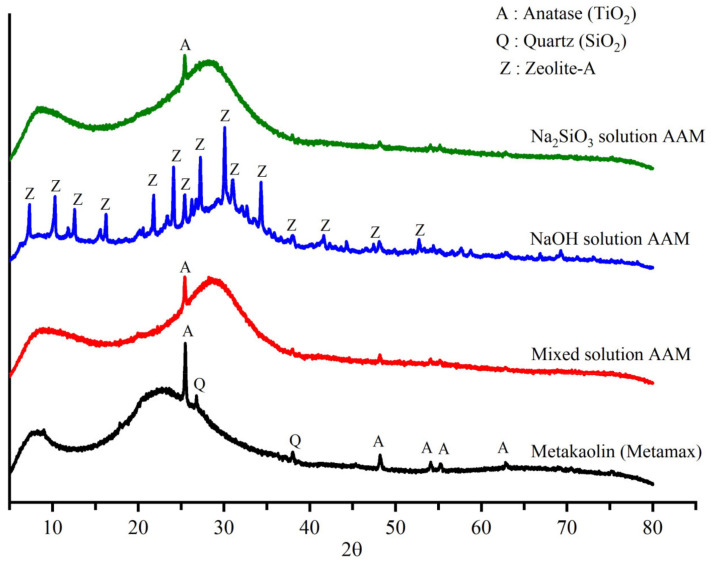
X-ray diffraction patterns of metakaolin and AAM samples produced using different alkali activators.

Figure 7 shows the XRD patterns of metakaolin, the mixed solution-based AAM, NaOH solution-based AAM, and Na_2_SiO_3_ solution-based AAM. Metakaolin contained an amorphous phase, as indicated by the broad peak in the 2θ range from 15° to 30° and crystalline phases of quartz and titanium dioxide (anatase phase). Once metakaolin was activated with a mixed solution of NaOH and Na_2_SiO_3_ or a solution of Na_2_SiO_3_, the amorphous phase of the metakaolin started to dissolve to form a new amorphous alkali silicate network, as indicated by the shift of the peak in the 2θ range from 23° to 30°. The XRD pattern of the NaOH solution-based AAM showed that its amorphous phase had shifted to 30° and that a new peak representing the zeolite-A phase was present. The results of the Rietveld analysis of metakaolin and the AAM samples are listed in Table 3. The amorphous phase represented 98.20% of metakaolin accompanied by small amounts of quartz and anatase phase. Following the polymerization reaction, the amorphous phases of the mixed solution-based AAM and Na_2_SiO_3_ solution-based AAM reached 99.6% with a small amount of quartz and anatase crystalline phase. In the NaOH solution-based AAM, the metakaolin also was dissolved, and a new zeolite phase appeared (approximately 3.8%) after 28 days of reaction. Moreover, quartz and anatase phase still appeared in the AAM but could not be observed in the XRD pattern (Figure 7) due to the overlapping of the peaks.

### 3.3. SEM-EDX Analysis

**Figure 8:**
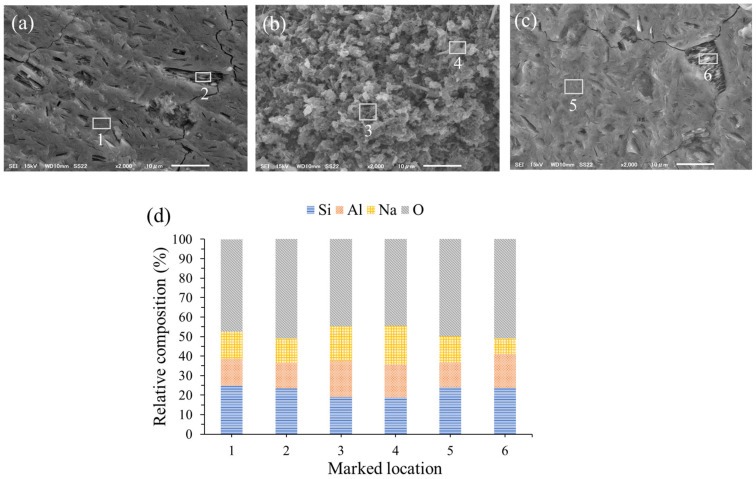
Scanning electron microscopy images of (**a**) the mixed solution-based AAM, (**b**) the NaOH solution-based AAM, (**c**) the Na_2_SiO_3_ solution-based AAM, and (**d**) energy dispersive X-ray analysis results.

Information about the morphology and elemental composition of the AAMs was obtained using SEM and EDX. The significantly different morphology of AAMs at curing times of 28–180 days was not observed. Figure 8 displays the SEM images of the AAM samples at 84 days and the EDX results, confirming the elemental compositions at the locations marked. The morphology of the fracture surface of the mixed solution-based AAM (Figure 8a) shows unreacted metakaolin particles with a sheet structure and a Si/Al ratio of 1.79 (Location 2), and a homogenous surface structure with a Si/Al ratio of 1.80 (Location 1). As shown in Figure 8c, when a Na_2_SiO_3_ solution was used as the activator, a homogenous and dense structure (Si/Al = 1.85, Location 5) could be observed with a few unreacted metakaolin particles (Si/Al = 1.36, Location 6). Figure 8b shows zeolite-A crystals in the sample, which was previously confirmed by XRD. The Si/Al ratios of the zeolite phase is in the 1.02–1.11 range (Locations 3 and 4), which is consistent with the zeolite-A formula (Na_2_O∙ Al_2_O_3_∙ 2SiO_2_∙ 4-5H_2_O, Si/Al = 1) [28].

### 3.4. Compressive Strength and Bulk Density

**Figure 9:**
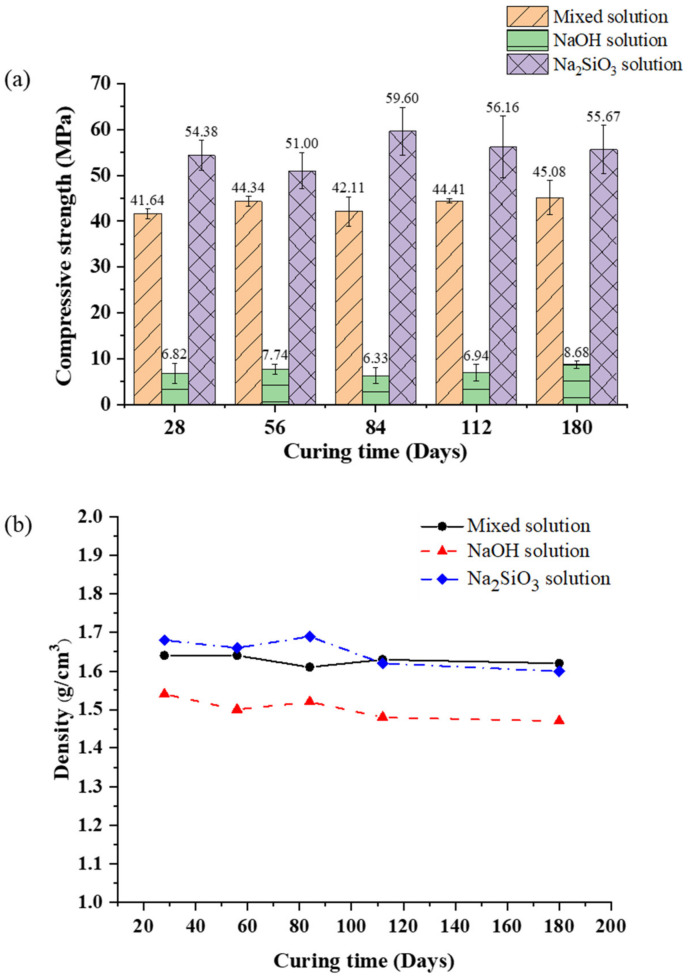
(**a**) Compressive strength and (**b**) bulk density of the mixed solution-based AAM, NaOH solution-based AAM, and Na_2_SiO_3_ solution-based AAM, for different curing times.

The mechanical properties of the AAM samples were investigated by studying their compressive strength and bulk density, as shown in Figure 9. The compressive strength of the mixed solution-based AAM was in the 41–45 MPa range for curing times of 28–180 days; it slightly increased when the sample was cured for a longer time. The bulk density of the mixed solution-based AAM ranged from 1.62 to 1.64 g/cm^3^. The compressive strength and bulk density of the NaOH solution-based AAM were the lowest at approximately 7–8 MPa and 1.47–1.54 g/cm^3^, respectively. These results indicate that the NaOH solution-based AAM samples had transformed into the zeolite phase, which was broken into a three-dimensional network of aluminosilicate chains. The compressive strength of the Na_2_SiO_3_ solution-based AAM sample for any curing age was higher than the compressive strength of the other two AAM samples. The Na_2_SiO_3_ solution-based AAM sample cured at 20 °C for 84 days had the highest compressive strength and bulk density of 59.60 MPa and 1.69 g/cm^3^, respectively. The SEM image of the Na_2_SiO_3_ solution-based AAM reveals few cracks and a dense structure, illustrating the reason for the highest bulk density and compressive strength of the Na_2_SiO_3_ solution-based AAM. Following 84 days of curing, the compressive strength of the Na_2_SiO_3_ solution-based AAM began to decrease slightly, reaching 56.16 MPa and 55.67 MPa after 112 and 180 days of curing, respectively. The compressive strength of the Na_2_SiO_3_ solution-based AAM decreased because water evaporation and structure formation were obstructed by the Na_2_SiO_3_ solution [10,29].

## 4. Discussion

The ATR-FTIR results of the AAM pastes and powder indicate that their polymerization reactions consist of two processes. In the first process, the aluminosilicate amorphous phase of the starting material dissolves, as indicated by the disappearance of the peaks of the mixed solution-based AAM paste and NaOH solution-based AAM paste at 1200 cm^−1^ and 1175 cm^−1^, respectively. This result indicates that the NaOH solution breaks the bridges of Si–O–Si and Si–O–Al. The Al–O bonding is easier to break than the Si-O bonding because its bonds are weaker than those of the Si-O bonding [27]. Moreover, the peak in the 1100–1000 cm^−1^ range shifted toward a lower wavenumber. This result can be explained by the disappearance of polysilicates, in favor of orthosialate [26]. The dissolution process consists of Na^+^ and OH^−^ reacting with metakaolin to form the primary unit of the orthosialate molecule, which has a Si–O–Na^+^ terminal bond [30]. The second process of the polymerization reaction consists of reorganization and condensation. Reorganization causes an increase in the peak between 980 and 950 cm^−1^, which can be attributed to the increase in the Si–O–Na^+^ dipole moment and the sharing of aluminum in the AAM materials [31]; this change in intensity can be seen in the spectra of all of the AAM pastes. The peaks between 880 and 850 cm^−1^ increased, indicating increased Si–O stretching and OH bending (Si–OH), caused by the condensation of the sialate groups on the aluminosilicate frameworks. A peak at 700 cm^−1^ (Al–O vibrations) could be observed in the AAM spectra. It indicates that sodium aluminate was formed after the AAMs were cured for 28 days [32].

**Figure 10:**
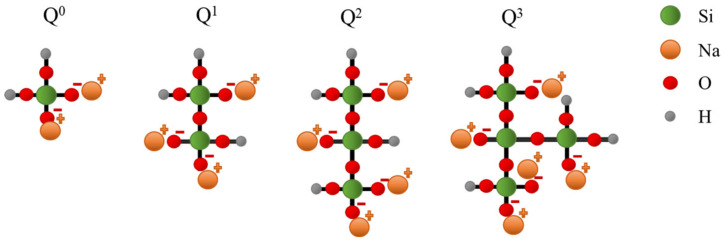
A unit of Q^0^, Q^1^, Q^2^, and Q^3^ silicate unit.

The reaction mechanisms of the AAMs prepared using the NaOH solution, Na_2_SiO_3_ solution, and a mixed solution of NaOH and Na_2_SiO_3_, identified from the ATR-FTIR spectra, can be explained as follows:

- Reaction mechanisms of NaOH based AAM; When the NaOH solution reacts with metakaolin, it acts as an activator that can dissolve the silica and alumina precursors in the metakaolin to form transient [SiO_4_]^4−^ and [AlO_4_]^5−^ nuclei; these nuclei react and grow in an orderly manner to form zeolite crystals with no change in their compositions [33,34];
- The reaction mechanism of the Na_2_SiO_3_ solution with metakaolin; Sodium silicate functions as an activator, and a variety of silicate units (Q^0^, Q^1^, Q^2^, and Q^3^) act as a template for Al–OH condensation from [AlO_4_]^5^, which comes from metakaolin [33,35,36]. The AAM synthesized from sodium silicate tends to be amorphous. The reaction between metakaolin and the Na_2_SiO_3_ solution occurs slowly and forms large oligomers;
- A unit of Q^0^, Q^1^, Q^2^, and Q^3^ silicate unit (Figure 10):
- The reaction mechanism of the mixed solution with metakaolin; When NaOH and Na_2_SiO_3_ solutions are used together as activators, their functions become supplementary. Na_2_SiO_3_ solution can dominate the reaction mechanism because Na_2_SiO_3_ slowly reacts. Therefore, the dissolution of [AlO4]^5−^ species is accelerated by the NaOH solution during the early period of the reaction. Then, Na_2_SiO_3_ reacts and forms a larger network and eventually a 3D network of AAM through a condensation reaction.

For XRD and SEM images of the NaOH solution-based AAM analyses, the results showed that zeolite-A was produced in the sample at 28 days. Moreover, the zeolite phase and structure of zeolite are related to its mechanical properties which had a low strength and density. In the Na_2_SiO_3_ solution and mixed solution-based AAM, only the amorphous phase and impurity from raw materials were displayed with the shift of the hump peak to a higher 2θ position. This result indicated that the new amorphous phase occurred after the alkali activation reaction. The participation of an amorphous phase without a new phase led to the high strength of the binder. The large oligomer structure of the AAMs by the Na_2_SiO_3_ solution has a significant effect on the density and mechanical strength development in the sample. However, the strength of the sample could be decreased when the long chain structure of oligomers was attacked. In addition, the compressive strength of the mixed solution-based AAM was more stable than the Na_2_SiO_3_ solution-based AAM because of a 3D network, composed of various unit kinds of connected SiO_4_ and AlO_4_ in the sample.

## 5. Conclusions

ATR-FTIR spectroscopy was successfully used to observe the polymerization kinetics of AAMs prepared using different alkali activator solutions. Polymerization could be investigated using a functional group analysis of the intensity and wavenumber changes of the Si–O–M bonds (M = Si, Al, or Na) because the main networks of the AAMs are formed by Si–O–M bonds. The results of the analysis indicated that Si and Al from metakaolin rapidly dissolved in the NaOH solution, resulting in polymerization to form crystalline zeolite. The Na_2_SiO_3_ solution slowly reacted with metakaolin and acted as a template for the aluminosilicate framework. The mixed solution of NaOH and Na_2_SiO_3_ reacted with metakaolin and simultaneously formed an aluminosilicate network.

Different tests were performed to investigate the effect of alkali activators on the microstructure and mechanical properties of alkali-activated metakaolin. Sodium silicate solution-activated metakaolin had the highest structural density and compressive strength due to the chemical and physical bonding of the solution with aluminosilicate. When the metakaolin was activated with a NaOH solution, zeolite-A was formed, which had inferior mechanical properties. The mixed solution-based AAM had more stable mechanical properties than the Na_2_SiO_3_ solution-based AAM when the samples were cured for a long time. Thus, the alkali activators and silicate and hydroxide ratios used in the preparation of AAMs play an important role in controlling the reaction mechanisms, which determine the chemical and physical properties of the prepared AAMs.

## Figures and Tables

**Table 1:** Mol ratios of alkali-activated materials (AAMs) obtained using different alkali activators.

Mix Code	Mol Ratio			Mass Ratio (wt%)
	Si/Al	Na/Al	H_2_O/Na_2_O	Liquid/Solid
Mixed solution-based AAM	1.77	1.17	13	1.90
NaOH solution-based AAM	1.00	1.17	13	1.50
Na_2_SiO_3_ solution-based AAM	2.26	1.17	13	2.16

**Table 2:** Characteristic infrared stretching vibrational bands of a metakaolin-based AAM.

Wavenumber (cm^−1^)	Bonding
1250–1200	Asymmetric stretching (Si–O–Si and Al–O–Si)
1160–1105	Asymmetric stretching (Si–O–Si)
1060–1000	Asymmetric stretching (Si–O–Si and Al–O–Si)
980–950	Si-O stretching (Si–O–Na, K or Li)
880–850	Si-O stretching and OH bending (Si-OH)
810–800	Al–O bending vibrations
700	Asymmetric stretching (Si–O–Si and Al–O–Si)

**Table 3:** Results of the Rietveld analysis of metakaolin and AAM samples produced using different alkali activators.

Phase	Raw Material	AAM Sample		
	Metakaolin (Metamax)	Mixed Solution	NaOH Solution	Na_2_SiO_3_ Solution
Quartz	0.30 ± 0.43	<0.1	0.20 ± 0.43	<0.1
Anatase	1.60 ± 0.29	0.4 ± 0.12	0.50 ± 0.36	0.4 ± 0.12
Zeolite-A	-	-	3.8 ± 0.29	-
Amorphous	98.20 ± 0.48	99.6 ± 0.05	95.5 ± 0.11	99.6 ± 0.28

## Data Availability

Not applicable.

## References

[1] Wang A., Zheng Y., Zhang Z., Liu K.L., Li Y., Shi L., Sun D. (2020). The Durability of Alkali-Activated Materials in Comparison with Ordinary Portland Cements and Concretes: A Review. Res. Civil Eng..

[2] Palomo A., Grutzeck M., Blanco M. (1999). Alkali-activated fly ashes: A cement for the future. Cem. Concr. Res..

[3] Ayeni O., Onwualu A.P., Boakye E. (2021). Characterization and mechanical performance of metakaolin-based geopolymer for sustainable building applications. Constr. Build. Mater..

[4] Karakoç M.B., Türkmen İ., Maraş M.M., Kantarci F., Demirboğa R., Toprak M.U. (2014). Mechanical properties and setting time of ferrochrome slag based geopolymer paste and mortar. Constr. Build. Mater..

[5] Ryu G.S., Lee Y.B., Koh K.T., Chung Y.S. (2013). The mechanical properties of fly ash-based geopolymer concrete with alkaline activators. Constr. Build. Mater..

[6] Natassia Bratti da Silva N., Danila Ferreira N., Adriano Michael B. (2021). Valorization of rice husk ash and aluminum anodizing sludge as precursors for the synthesis of geopolymers. J. Clean. Prod..

[7] Onutai S., Jiemsirilers S., Thavorniti P., Kobayashi T. (2015). Aluminium hydroxide waste based geopolymer composed of fly ash for sustainable cement materials. Constr. Build. Mater..

[8] Król M., Rożek P., Chlebda D., Mozgawa W. (2018). Influence of alkali metal cations/type of activator on the structure of alkali-activated fly ash–ATR-FTIR studies. Spectrochim. Acta A Mol. Biomol. Spectrosc..

[9] Davidovits J. (1991). Geopolymers. J. Therm. Anal..

[10] Khale D., Chaudhary R. (2007). Mechanism of geopolymerization and factors influencing its development: A review. J. Mater. Sci..

[11] Duxson P., Fernández-Jiménez A., Provis J.L., Lukey G., Palomo A., van Deventer J.S.J. (2007). Geopolymer technology: The current state of the art. J. Mater. Sci..

[12] Palomo Á., Alonso S., Fernandez-Jiménez A., Sobrados I., Sanz J. (2004). Alkaline activation of fly ashes: NMR study of the reaction products. J. Am. Ceram. Soc..

[13] Lee W., van Deventer J. (2002). Structural reorganisation of class F fly ash in alkaline silicate solutions. Colloids Surf. A Physicochem. Eng. Asp..

[14] Cundy C.S., Cox P.A. (2005). The hydrothermal synthesis of zeolites: Precursors, intermediates and reaction mechanism. Microporous Mesoporous Mater..

[15] Matsuda A., Maruyama I., Meawad A., Pareek S., Yoshikazu A. (2019). Reaction, Phases, and Microstructure of Fly Ash-Based Alkali-Activated Materials. J. Adv. Concr. Technol..

[16] Guo B., Pan D., Liu B., Volinsky A.A., Fincan M., Du J., Zhang S. (2017). Immobilization mechanism of Pb in fly ash-based geopolymer. Constr. Build. Mater..

[17] Rees C.A., Provis J.L., Lukey G.C., van Deventer J.S.J. (2007). In Situ ATR-FTIR Study of the Early Stages of Fly Ash Geopolymer Gel Formation. Langmuir.

[18] Hajimohammadi A., Provis J.L., van Deventer J.S.J. (2010). Effect of Alumina Release Rate on the Mechanism of Geopolymer Gel Formation. Chem. Mater..

[19] Chen X., Meawad A., Struble L. (2014). Method to Stop Geopolymer Reaction. J. Am. Ceram. Soc..

[20] Pavel R. (2010). Effect of curing temperature on the development of hard structure of metakaolin-based geopolymer. Constr. Build. Mater..

[21] Gharzouni A., Samet B., Baklouti S., Joussein E., Rossignol S. (2016). Addition of low reactive clay into metakaolin-based geopolymer formulation: Synthesis, existence domains and properties. Powder Technol..

[22] Autef A., Prud’Homme E., Joussein E., Gasgnier G., Pronier S., Rossignol S. (2013). Evidence of a gel in geopolymer compounds from pure metakaolin. J. Solgel Sci. Technol..

[23] Lee W.K.W., van Deventer J.S.J. (2003). Use of Infrared Spectroscopy to Study Geopolymerization of Heterogeneous Amorphous Aluminosilicates. Langmuir.

[24] Chindaprasirt P., Jaturapitakkul C., Chalee W., Rattanasak U. (2009). Comparative study on the characteristics of fly ash and bottom ash geopolymers. Waste Manag..

[25] Rattanasak U., Chindaprasirt P. (2009). Influence of NaOH solution on the synthesis of fly ash geopolymer. Miner. Eng..

[26] Król M., Rożek P., Chlebda D., Mozgawa W. (2019). ATR/FT-IR studies of zeolite formation during alkali-activation of metakaolin. Solid State Sci..

[27] Akolekar D., Chaffee A., Howe R.F. (1997). The transformation of kaolin to low-silica X zeolite. Zeolites.

[28] Kumar N., Tiwari K., Meenu K., Sharma A., Jain A., Singh S., Tomar R. (2019). Utilization of Various Analogy of Synthetic Nanoporous Zeolites and Composite of Zeolites for Decontamination/Detoxification of CWA Simulants—An Updated Review. Int. J. Nonferrous Metall..

[29] Cheng T., Chiu J. (2003). Fire-resistant geopolymer produced by granulated blast furnace slag. Miner. Eng..

[30] Ralli Z., Pantazopoulou S. (2021). State of The Art on Geopolymer ConcreteInt. J. Struct. Integr..

[31] Fernández-Jiménez A., Palomo A. (2005). Mid-infrared spectroscopic studies of alkali-activated fly ash structure. Microporous Mesoporous Mater..

[32] Watling H. (1998). Spectroscopy of Concentrated Sodium Aluminate Solutions. Appl. Spectrosc..

[33] Sagoe-Crentsil K., Weng L. (2007). Dissolution processes, hydrolysis and condensation reactions during geopolymer synthesis: Part II. High Si/Al ratio systems. J. Mater. Sci..

[34] Rocha J., Klinowski J., Adams J.M. (1991). Synthesis of zeolite Na-A from metakaolinite revisited. J. Chem. Soc. Faraday Trans..

[35] Singh P.S., Trigg M., Burgar I., Bastow T. (2005). Geopolymer formation processes at room temperature studied by ^29^Si and ^27^Al MAS-NMR. Mater. Sci. Eng. A..

[36] Barbosa V., MacKenzie K., Thaumaturgo C. (2000). Synthesis and Characterisation of Materials Based on Inorganic Polymers of Alumina and Silica: Sodium Polysialate Polymers. Int. J. Inorg. Mater..

